# Short-Term Performance Effects of Three Different Low-Volume Strength-Training Programmes in College Male Soccer Players

**DOI:** 10.2478/hukin-2014-0014

**Published:** 2014-04-09

**Authors:** João Brito, Fabrício Vasconcellos, José Oliveira, Peter Krustrup, António Rebelo

**Affiliations:** 1 National Sports Medicine Programme, Excellence in Football Project, Aspetar – Qatar Orthopaedic and Sports Medicine Hospital, Qatar.; 2 Centre of Research, Education, Innovation and Intervention in Sport, Faculty of Sport, University of Porto, Portugal.; 3 Rio de Janeiro State University, Brazil.; 4 Research Centre in Physical Activity, Health and Leisure, Faculty of Sport, University of Porto, Portugal.; 5 Sport and Health Sciences, St. Luke’s Campus, College of Life and Environmental Sciences, University of Exeter, United Kingdom.; 6 Department of Nutrition, Exercise and Sports, Section of Human Physiology, University of Copenhagen, Denmark.

**Keywords:** soccer, resistance training, plyometric training, complex and contrast training

## Abstract

This study aimed to analyse the short-term performance effects of three in-season low-volume strength-training programmes in college male soccer players. Fifty-seven male college soccer players (age: 20.3±1.6 years) were randomly assigned to a resistance-training group (n=12), plyometric training group (n=12), complex training group (n=12), or a control group (n=21). In the mid-season, players underwent a 9-week strength-training programme, with two 20 min training sessions per week. Short-term effects on strength, sprint, agility, and vertical jump abilities were measured. All training groups increased 1-RM squat (range, 17.2–24.2%), plantar flexion (29.1–39.6%), and knee extension (0.5–22.2%) strength compared with the control group (p<0.05). The resistance-training group increased concentric peak torque of the knee extensor muscles by 9.9–13.7%, and changes were greater compared with the control group (p<0.05). The complex training group presented major increments (11.7%) in eccentric peak torque of the knee flexor muscles on the non-dominant limb compared with the control group and plyometric training group (p<0.05). All training groups improved 20-m sprint performance by 4.6–6.2% (p<0.001) compared with the control group. No differences were observed in 5-m sprint and agility performances (p>0.05). Overall, the results suggest that in-season low-volume strength training is adequate for developing strength and speed in soccer players.

## Introduction

During a typical soccer game, players perform 150–250 brief intense actions (Bangsbo et al., 2006), including changes in activity every 3–5 s, 30–40 sprints, 30–40 tackles and jumps (Mohr et al., 2003), decelerations, kicks, and dribbles. Soccer is becoming increasingly more athletic; hence, the contribution of strength, power, and their derivatives (acceleration, sprinting, and jumping) might be beneficial in many game situations (Hoff and Helgerud, 2004). Therefore, conditioning coaches feel the need to include ancillary strength-training sessions as part of routine football training programmes.

Traditional modalities to improve strength, include resistance training and plyometric exercises with movement patterns as close as possible to specific football skills, aiming to warrant the highest degree of transference between strength gains and soccer technical skills. For this purpose, conditioning coaches often refer to the complex training method, which combines weight lifting of heavy-loads with plyometric exercises, set for set, in the same workout (Robbins, 2005). The rational underlying this method is the theory of a post-activation potentiation of the neuromuscular system, i.e. a phenomenon induced by a voluntary conditioning contraction, typically performed at maximal or near-maximal intensities, that may increase peak force and the rate of force development during subsequent twitch contractions (Tillin and Bishop, 2009).

In soccer, many studies have shown that strength training combining weight lifting and plyometric exercises results in significant improvements in match-related physical abilities (Kotzamanidis et al., 2005; Maio Alves et al., 2010; Perez-Gomez et al., 2008). Notwithstanding, soccer is a team sport, so that the largest proportion of training practice is devoted to field-based conditioning drills to ensure the players preparedness for specific demands of the match play. This is a major issue during the competitive period, when players might have little time for ancillary strength training. Conditioning coaches might thereby feel the need to rationalise the time and volume devoted to strength-training programmes, as a strategy to guarantee that players accomplish with the prescribed training regimens.

In the present study, the short-term effects of three different in-season low-volume strength-training programmes on strength, sprint, agility, and vertical jump performance of soccer players were tested. It was examined whether adding plyometric-skill exercises to a programme with high-load weight training could be advantageous, compared to basic resistance training or plyometric training only.

## Material and Methods

### Participants

Fifty-seven adult male soccer players were invited to participate in the study. The participants were college students engaged in different local soccer clubs. All players were informed about the protocol, and signed an informed consent form before the investigation. The Scientific Board from the Faculty of Sport, University of Porto approved the design of the study. The players were then randomly assigned to 4 groups: resistance-training group (RT, n=12), plyometric-training group (PT, n=12), complex-training group (CT, n=12), and control group (CG; n=21). The groups were similar (p>0.05) in age (CG: 20.7±1.0 yrs; RT: 20.3±0.9 yrs; PT: 20.0±0.6 yrs; CT: 19.9±0.5 yrs), body mass (CG: 71.4±2.1 kg; RT: 72.8±1.8 kg; PT: 71.6±2.3 kg; CT: 72.2±1.1 kg), and body height (CG: 178±5 cm; RT: 176±5 cm; PT: 176±5 cm; CT: 180±7 cm). Furthermore, during pre-training, no statistical differences between the groups were observed with regard to any of the tests performed.

### Measures

All participants accomplished a 3-day testing set; the players were evaluated within 1 week, in different days interspersed by at least 48h. Primarily, the players were evaluated in one repetition maximum (1-RM) in the squat, knee extension, and plantar flexion exercises, aiming to determine maximal strength and to further prescribe the training workload. On the following day, the players were evaluated for isokinetic strength. The last day of testing was devoted to measure the squat jump (SJ) and countermovement jump (CMJ), 5- and 20-m sprinting, and agility performance. The first evaluation was carried out before the start of the intervention programme, and the second after 9 weeks of training.

The determination of 1-RM was conducted according to the procedures suggested by Kraemer and Fry (1995). The evaluations were carried out after a 1-week familiarisation period, in which participants learned the exercise execution techniques. The participants were always kept under surveillance of one member of the research team.

Isokinetic assessment (Biodex, System IV, USA) included bilateral measurements of knee extensors (quadriceps, Q) and flexors (hamstrings, H). Measurements were preceded by a 5-min warm-up on a cycle ergometer and a specific sub-maximal protocol on the dynamometer in order to familiarize the participants with the isokinetic device and test procedure. Participants were tested in the seated position with the back inclined at 85º using stabilisation straps at the trunk, abdomen and thigh to prevent inadequate joint movements. The arms were held comfortably across the chest. The axis of the dynamometer lever arm was aligned with the distal point of the lateral femoral condyle. A range of knee motion of 90º (0º=full extension) was provided both for the concentric and the eccentric tests and the gravity correction procedure was employed. The testing protocol consisted of concentric actions of both quadriceps (Q) and hamstrings (H) at 60º/s (3 repetitions). Afterwards, the hamstring muscles were tested in the eccentric mode at 60º/s (3 repetitions). Testing sets were separated by a 1-min rest interval. During the test, oral and visual feedback was given. The concentric H:Q peak torque ratio (conventional H/Q ratio) and the eccentric hamstrings:concentric quadriceps peak torque ratio (functional H/Q ratio) were calculated.

In the SJ, participants performed a maximal vertical jump with hands on the waist, starting from an angle of 90° at the knee; in the CMJ, the participants performed a maximal vertical jump starting from a standing position, with arm swing not allowed. All jumps were performed on a jump mat (Digitime 1000, Digitest, Finland). Participants performed 2 trials in each jump type, and the best result was used in further analysis.

Sprint and agility performance were evaluated outdoors, in an artificial turf ground, using photoelectric cells (Speed Trap II, Brower Timing Systems, USA). Sprint evaluation was accomplished through a flat sprint test that was carried out in a straight 20-m line. The times were measured through 3 pairs of photoelectric cells positioned at the starting line, at 5 and 20 m. The lower (fastest) time of 2 trials for each test was retained for analysis. Agility was evaluated by the T-test, as described by Semenick (1990). The subject began with both feet 30 cm behind the starting line (A). The player sprinted forward 10 m to point B and touched a marker (cone) with the right hand, then sprinted 5 m to the left and touched another marker (C) with the left hand, then sprinted 10 m to the right and touched a third marker (D) with the right hand, and finally sprinted back to point B and touched the marker with the left hand, after which he turned 90º, and returned to the starting point A running passed the finishing line. The photoelectric cells were placed at the starting/finishing line (A) to record the elapsed time. Players were instructed to run as fast as possible. The fastest time of two trials was retained for analysis.

### Procedures

The 9-week in-season strength training intervention programme was conducted in 3 experimental groups with two training sessions per week. Additionally, all groups performed their routine soccer training, based on technical and tactical drills, and small-sided games. The training programmes were adapted from Maio Alves et al. (2010). After a 10-min warm-up with light jogging or cycling, the resistance-training group (RT) performed high-load weight training, the plyometric-training group (PT) performed plyometric training without weight-bearing exercises, and the complex-training group (CT) performed high-load weight training followed by plyometric exercises, set by set. The exercises for each of the training groups were as described in Table 1. The control group performed the routine soccer training only.

The training sessions lasted 15–20 min (including the warm-up), and were organised in 3 stations. The players performed 1 set in each station. Training volume (i.e., total number of sets x repetitions in each set) was unaltered during the training period for all groups, but for RT and CT the load of the weight-bearing exercises was increased by 5% from 1-RM each 3 weeks. All training sessions were supervised by one of the investigators during the entire training period. None of the players had accomplished any strength-training regimen prior to the study. Therefore, two familiarisation training sessions to the strength training programme were granted, as to optimise exercise execution, prevent possible injuries, and attenuate the learning effect (Kotzamanidis et al., 2005).

### Statistical analyses

Data are presented as mean±SD, unless otherwise noted, or relative change after the intervention (%). Comparisons between groups in post-intervention performances in all tests were calculated by ANOVA, using the baseline value as the covariate to correct for any difference in groups at baseline. When significant differences were found, Bonferroni post hoc comparisons were used to identify between-group differences. Effect sizes were classified according to Hopkins (Hopkins, 2010) as trivial (*d* < 0.2), small (0.2 < *d* < 0.6) moderate (0.6 < *d* < 1.2), large (1.2 < *d* < 2.0), very large (2.0 < *d* < 4.0), nearly perfect (*d* > 4.0), and perfect (*d* = infinite). The level of statistical significance was set at p<0.05.

## Results

### Strength

The training-related changes in 1RM, concentric and eccentric strength are presented in Table 2. All training groups increased 1-RM squat, knee extension, and plantar flexion strength compared with the CG (p<0.001).

The RT increased concentric peak torque of the knee extensor muscles in the dominant limb compared to the CG and PT [RT (13.7%) vs. CG (1.2%) and PT (−2.2%); p<0.01], with intermediate values for the CT (11.7%). No significant changes were observed for the non-dominant limb (p=0.064). The RT also increased concentric peak torque of the knee flexor muscles on the dominant limb compared with the CG (9.9% vs. 0.1%; p=0.010); intermediate values were observed for the PT and CT (4.6 and 4.3%, respectively). No significant changes were observed for the non-dominant limb (p=0.318).

The RT and CT elevated by 8.6% and 7.4%, respectively, eccentric peak torque of the knee flexor muscles on the dominant limb (CG, 1.4%; PT, 0.5%), but post-intervention differences were not significant between groups (p=0.077). On the non-dominant limb, CT increased eccentric peak torque comparing with CG and PT [CT (11.7%) vs. CG (−0.7%) and PT (1.4%); p<0.05], with intermediate values for RT (5.1%). The conventional and functional H/Q ratios were not affected over the training period in any of the training groups (p>0.05).

### Speed, agility and jumping performance

**Figure 1.**
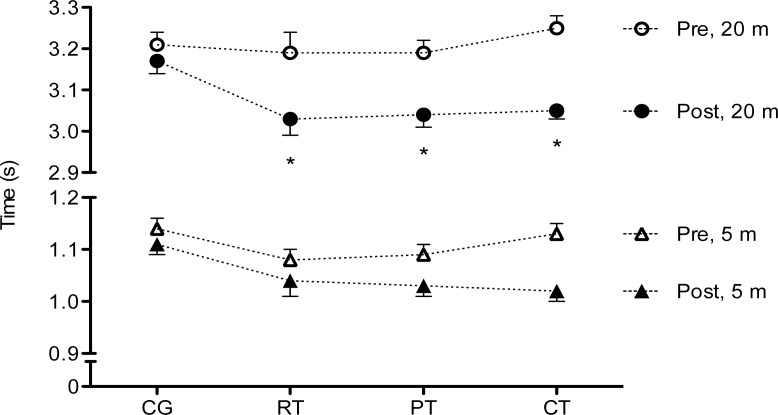
Changes in 5- and 20-m sprint in the control group (CG, n=21), resistance training (RT, n=12), plyometric training (PT, n=12), and complex training (CT, n=12) groups after 9 weeks of training

Enhancements in 20-m sprint performance were significantly different between all training groups and the CG (CG, 3.21±0.13 vs. 3.17±0.12 s; RT, 3.19±0.18 vs. 3.02±0.16 s; PT, 3.19±0.09 vs. 3.04±0.10 s; CT, 3.25±0.09 vs. 3.05±0.07 s; F=8.375; p<0.001; η^2^=0.344; Figure 1). No significant post-intervention differences were observed in 5-m sprint performance (F=1.784; p=0.163; η^2^=0.100; Figure 1), nor in the agility test (F=1.958; p=0.132; η^2^=0.103).

Over the training period, no changes were observed between groups in SJ (F=1.519; p=0.222; η^2^=0.087), and CMJ (F=0.823; p=0.488; η^2^=0.049) performances.

## Discussion

In the present study, college male soccer players performed low-volume strength training sessions twice a week throughout 9 weeks. The training sessions comprised one set of three exercises. Given the low volume of training, the magnitude of strength enhancements observed in the present study was rather unforeseen; all training regimens were effective in improving muscle strength and 20-m sprint performance. The results also indicated that complex training might be effective in developing eccentric strength. However, no significant differences were observed between the three training modalities in maximal strength, sprint, agility and jump performances over the training period.

An increase in maximal strength is usually associated with improvements in relative strength and in power-related abilities (Hoff, 2005; Markovic and Mikulic, 2010; Ronnestad et al., 2008). In the present study, changes in 1-RM squat, plantar flexion, and knee extension were significant in all trained groups. The results were already expected for the RT and CT, but such improvements in maximal strength were actually surprising within the PT. The positive increments in lower-extremity maximal strength might be related with improvements in neuromuscular function (e.g. increased neural drive to the agonist muscles; changes in muscle activation strategies) that are likely to occur in response to plyometric training (Markovic and Mikulic, 2010). Notwithstanding, players were only performing one set for each plyometric exercise drill; this is a very low-volume plyometric training compared to previous studies (Chelly et al., 2010; Impellizzeri et al., 2008; Sedano et al., 2011; Thomas et al., 2009).

The RT also improved peak torque of the knee extensor muscles. In soccer, the quadriceps muscle group is important for jumping and ball kicking. Therefore, the results of the present study highlight that heavy-loaded squat and knee extensor exercises—emphasising maximal mobilization of force in the concentric action—should be integrated as part of ancillary conditioning programmes for soccer players. Nonetheless, taking into account the importance of hamstring strengthening for reducing of the risk of injury in soccer players (Croisier et al., 2008), a limitation of the current strength training programmes would be the lack of exercises focusing specifically on the hamstrings. However, the RT increased concentric peak torque of the hamstrings on the dominant limb, whereas the CT improved eccentric peak torque of the knee flexor muscles on the non-dominant limb, although none of the weight-bearing exercises performed by the RT and PT focused on the posterior muscles of the thigh. Studies investigating the effectiveness of hamstring strength training programmes that combine plyometric drills with concentric and eccentric resistance training exercises are thereby needed.

It should be noted that, in soccer, the gains in muscular strength should not compromise speed of movement. In fact, the ultimate goal of strength training is to increase muscle strength so that acceleration and speed in soccer-specific skills, such as turning, sprinting and changing direction, may be enhanced (Bangsbo et al., 1991). In the present study, no significant changes were observed between the training groups and controls in agility and jump performances. However, players from all training groups improved 20-m sprint performance; this has been previously observed with other strength training programmes (Chelly et al., 2009; Chelly et al., 2010; Kotzamanidis et al., 2005; Maio Alves et al., 2010). Given that sprint performance is determined by acceleration and maximal velocity, the results of the present study suggest that college soccer players might benefit from strength training as a strategy to develop speed of movement.

## Conclusions

Soccer-specific plyometric skills can thereby be included into low-volume resistance training sessions, as a mean to warrant transference between the strength gains and technical skills. Given the small effect sizes observed in the current study, it should be noted that the sample size might be statistically too small to detect differences between all training regiments. No significant short-term differences were observed between the CT and RT over the training period. Also, the changes in strength and sprint performance observed due to plyometric training were rather unforeseen, given the very low-volume of training. In soccer, the time available for in-season ancillary strength training might be reduced to the “minimum possible volume”. Conditioning coaches need to optimise strength-training programmes, by using the best strategies to warrant transference between strength enhancements and match-specific technical skills. In the present study, we observed that combining high-load strength training with soccer-specific movements might be an effective strategy to improve strength and speed. Additionally, conditioning coaches should take into account that during the competitive season low-volume strength training sessions might grant performance-enhancing effects in college soccer players.

## Figures and Tables

**Table 1:** Contents of resistance training (RT), plyometric training (PT), and complex training (CT) sessions

	RT	PT	CT
Station 1	6 RPS of 85% of 1RM squat at 90°.	1 set of high skipping, cyclically, with thighs parallel to the ground, keeping a frequency of movement as high as possible during 5 m; 1 straight-line 5-m sprint.	RT + PT
Station 2	6 RPS at 90% of 1RM calf extension exercise in a leg press device.	8 maximal vertically jumps, trying to minimize ground contact time; 3 ball headers, jumping as high as possible.	RT + PT
Station 3	6 RPS at 80% of 1RM of leg extension.	6 maximal vertical jumps from the seated position on a stool, trying to reach the highest point; 3 drop jumps (60 cm), leaving for a vertical jump, executing a soccer heading, and trying to minimize ground contact time and maximize jump height.	RT + PT

RT+PT, contents of resistance training plus contents of plyometric training

**Table 2:** Pre- to post-intervention changes in strength profiles (1RM and peak torque) of soccer players performing resistance-training (RT), plyometric-training (PT), and complex-training (CT), as well as a control group (CG). Values are presented as mean±SD

	CG		RT		PT		CT		p	η^2^
	Pre	Post	Pre	Post	Pre	Post	Pre	Post		
**1RM**										
Squat	126 (20)	129 (22)	136 (24)	167(29)	127 (23)	149 (19)	120 (14)	149 (20)	<0.001	0.41^*^
Plantar flex _Dom_	110 (26)	114 (29)	105 (19)	146 (24)	108 (17)	141 (30)	112 (37)	156 (29)	<0.001	0.44^*^
Plantar flex _Ndom_	107 (27)	113 (30)	104 (19)	142 (28)	105 (19)	136 (30)	110 (41)	153 (31)	<0.001	0.36^*^
Knee ext _Dom_	57 (11)	57 (11)	58 (12)	71 (9)	60 (9)	68 (13)	58 (8)	67 (9)	<0.001	0.49^*^
Knee ext _Ndom_	58 (11)	58 (11)	58 (12)	71 (12)	60 (10)	68 (11)	58 (9)	68 (10)	<0.001	0.48^*^
**Peak**										
**Torque**										
Q_con Dom_	204 (28)	207 (27)	205 (32)	233 (26)	221 (32)	216 (27)	206 (27)	230 (35)	<0.001	0.35^±^
Q_con Ndom_	201 (26)	199 (24)	193 (37)	206 (27)	213 (39)	217 (33)	195 (29)	211 (33)	0.064	0.14
H_con Dom_	114 (12)	114 (15)	116 (11)	130 (14)	127 (22)	130 (13)	119 (10)	123 (20)	0.014	0.21^#^
H_con Ndom_	113 (16)	112 (16)	110 (9)	116 (16)	125 (21)	125 (18)	115 (18)	119 (16)	0.318	0.07
H_ecc Dom_	185 (33)	187 (33)	190 (32)	207 (33)	205 (28)	206 (27)	191 (34)	205 (33)	0.077	0.13
H_ecc Ndom_	192 (30)	191 (29)	192 (33)	201 (33)	209 (39)	206 (38)	196 (28)	218 (35)	0.005	0.23^§^

Calf ext, calf extension; Leg ext, leg extension; con, concentric; ecc, eccentric; Dom, dominant limb; Ndom, non-dominant limb.

* RT,PT,CT>CG;

± RT>CG,PT;

# RT>CG;

§ CT>CG
